# Impact of 12 Weeks of Vitamin D_3_ Administration in Parkinson’s Patients with Deep Brain Stimulation on Kynurenine Pathway and Inflammatory Status

**DOI:** 10.3390/nu15173839

**Published:** 2023-09-02

**Authors:** Zofia Kinga Bytowska, Daria Korewo-Labelle, Konrad Kowalski, Witold Libionka, Katarzyna Przewłócka, Wojciech Kloc, Jan Jacek Kaczor

**Affiliations:** 1 Division of Bioenergetics and Physiology of Exercise, Faculty of Health Sciences, Medical University of Gdansk, 80-211 Gdansk, Poland; zofia.bytowska@gumed.edu.pl (Z.K.B.); k.przewlocka@gumed.edu.pl (K.P.); 2 Department of Physiology, Faculty of Medicine, Medical University of Gdansk, 80-211 Gdansk, Poland; daria.korewo-labelle@gumed.edu.pl; 3 Masdiag-Diagnostic Mass Spectrometry Laboratory, Stefana Żeromskiego 33, 01-882 Warsaw, Poland; konrad.kowalski@masdiag.pl; 4 Department of Neurosurgery, University Clinical Centre in Gdansk, 80-952 Gdansk, Poland; wlibionka@uck.gda.pl; 5 Department of Neurosurgery, Copernicus Medical Center, 80-803 Gdansk, Poland; wojciech.kloc@uwm.edu.pl; 6 Department of Psychology and Sociology of Health and Public Health, University of Warmia and Mazury in Olsztyn, 10-719 Olsztyn, Poland; 7 Department of Animal and Human Physiology, Faculty of Biology, University of Gdansk, 80-309 Gdansk, Poland

**Keywords:** Parkinson’s disease, deep brain stimulation, vitamin D, kynurenine pathway, inflammation

## Abstract

The current study aimed to investigate whether a 12-week Body Mass Index (BMI)-based (the higher the BMI, the higher the dosage) vitamin D_3_ administration may affect both the kynurenine pathway (KP) and the inflammatory state in Parkinson’s disease (PD) patients with deep brain stimulation (DBS) and may be useful for developing novel therapeutic targets against PD. Patients were randomly assigned to two groups: supplemented with vitamin D_3_ (VitD, n = 15) and treated with vegetable oil (PL, n = 21). Administration lasted for 12 weeks. The isotope dilution method by LC-MS/MS was applied to measure KP and vitamin D metabolites. Serum concentrations of cytokines such as IL-6 and TNF-α were measured using ELISA kits. After administration, the serum concentration of TNF-α decreased in PD patients with DBS. Moreover, in KP: 3-hydroksykynurenine (3-HK) was increased in the PL group, picolinic acid was decreased in the PL group, and kynurenic acid tended to be higher after administration. Furthermore, a negative correlation between 3-HK and 25(OH)D_3_ and 24,25(OH)_2_D_3_ was noticed. Our preliminary results provide further evidence regarding a key link between the KP substances, inflammation status, and metabolites of vitamin D in PD patients with DBS. These findings may reflect the neuroprotective abilities of vitamin D_3_ in PD patients with DBS.

## 1. Introduction

The population of people ages 65 and above is widely associated with neurodegenerative disorders, the second most widespread being Parkinson’s disease (PD). According to World Health Organization, the number of patients suffering from this disease was over 8.5 million in 2019. It is marked by motor symptoms including resting tremor, bradykinesia, and rigidity as well as non-motor symptoms, such as dementia, depression, sleep disturbances, among others. During disease progression, dopamine production drops due to the degeneration of substantia nigra pars compacta [1,2]. Although the disease is incurable, effective symptomatic therapies exist. Pharmacological treatment usually includes dopaminergic medication, with the precursor of dopamine, L-dihydroxyphenylalanine (L-dopa), being the most popular drug [3]. When medical therapy becomes ineffective, patients undergo deep brain stimulation (DBS) surgery. This type of treatment improves the motor symptoms, allowing for a reduction in the drug dosage [4].

Vitamin D is often described as one of the most relevant vitamins in the human body. It plays multiple roles, e.g., maintaining the calcium and phosphate balance, helping the immune system to differentiate, and increasing bone mineralization [5,6]. The synthesis of vitamin D_3_ (cholecalciferol) runs from 7-dehydrocholesterol percutaneously following sun exposure, followed by hydroxylation to 25-hydroxycholecalciferol (25(OH)D_3_) occurring in the liver. This is the inactive metabolite widely used to assess deficiency due to the prolonged persistence in the blood, contrary to the active form, 1,25-dihydroxycholecalciferol (1,25(OH)_2_D_3_), produced by the enzyme 1α-hydroxylase. Vitamin D deficiency is common at our latitude (49°00′–54°50′ N, 14°07′–24°09′ E) in Poland, Europe, a result of the limited number of sunny days during the year, particularly during the autumn-winter season [6,7,8]. PD patients have lower concentrations of vitamin D than their healthy age-matched controls; therefore, supplementation becomes essential [9]. Vitamin D deficiency is detrimental for humans, including the increased risk of developing depression, muscle atrophy, and possibly undergoing falls [10,11].

Supplementation of vitamin D_3_ is not popular among patients with PD. It is already known that PD patients suffer from a deficiency of vitamin D and have lower concentrations than healthy age-matched controls. Therefore, the doses for patients with PD should be expanded versus those for healthy people. In this study, we propose Body Mass Index (BMI)-based administration. A higher BMI is often associated with a higher content of body fat, which influences the distribution of vitamin D. Fat reduces the beneficial actions of vitamins [12].

Research on the supplementation of vitamin D_3_ in PD patients is scarce, especially those with implanted DBS. The dosage in the available research is determined for healthy people [13]. In our previous study, we took into account that the content of fat affects vitamin D metabolism, and therefore determined the doses based on the patient’s BMI [14].

The main route of tryptophan metabolism is the kynurenine pathway (KP). It is activated by indoleamine 2,3-dioxygenase (IDO) and tryptophan 2,3-dioxygenase (TDO). Tryptophan is converted to N-formyl-kynurenine and then quickly to kynurenine (KYN) due to N-formyl-kynurenine’s instability. Subsequently, KYN may be catabolized to 3-hydroksykynurenine (3-HK), kynurenic acid (KYNA), and anthranilic acid (AA). KYNA has neuroprotective properties. 3-HK, which is neurotoxic, is transformed into 3-hydroxyantrhranilic acid (3-HANA) and then catabolized to quinolinic acid (QUIN) or picolinic acid (PA), the first of which is neurotoxic, and the second may be neuroprotective. 3-HK may also be converted to xanthurenic acid (XANA) which also has neurotoxic properties. The kynurenine pathway is often disturbed in PD [15,16,17,18,19].

In PD patients, the markers of inflammation are often elevated compared with the healthy age-matched controls [20]. In a study conducted in 2019, the authors found that plasma concentration of tumour necrosis factor-alpha (TNF-α) was relevantly higher in PD patients than in controls [21]. Another study showed higher levels of pro-inflammatory cytokines such as interleukin-1β, interleukin-2, and interleukin-6 (IL-6) in the PD group compared to the control group. Furthermore, the level of anti-inflammatory cytokine interleukin-10 was lower in the PD group, although the change was not statistically relevant [22].

In our previous study [14], we demonstrated that BMI-based vitamin D_3_ administration improved physical performance in PD patients undergoing deep brain stimulation. Based on these findings, the current study was designed to explore whether vitamin D_3_ administration may affect both the kynurenine pathway and the inflammatory state in PD patients with DBS and may be useful for developing novel therapeutic targets against PD, which are independent of the dopaminergic system.

## 2. Materials and Methods

### 2.1. Design of the Study

This study was a randomized, double-blind, placebo-controlled clinical trial (NCT04768023) with 12 weeks of vitamin D_3_ administration in patients with PD who underwent DBS. The research has been approved by the Independent Bioethics Committee for Scientific Research at the Medical University of Gdansk, the number of approval: NKBBN/522-648/2019, date of approval: 3 December 2019. The procedures used in this study adhere to the tenets of the Declaration of Helsinki. All patients signed the consent to participate in the study and were informed of their right to resign at any point during the trial. The recruitment took place at the Neurosurgery Unit of the Nicholas Copernicus Hospital in Gdansk. Patients were divided into two groups in a 1:1 ratio to receive vitamin D_3_ (VitD) or placebo (PL). An Excel random number generator was used for the randomization. Vitamin D_3_ and placebo were placed in identical bottles labeled only with numbers and featuring no additional information. Product allocation and randomization were conducted by an independent researcher. Personnel involved in the data analysis and patients stayed blind until the database was examined for analysis. When all the data were analyzed the unblinding occurred.

### 2.2. Participants

**Figure 1:**
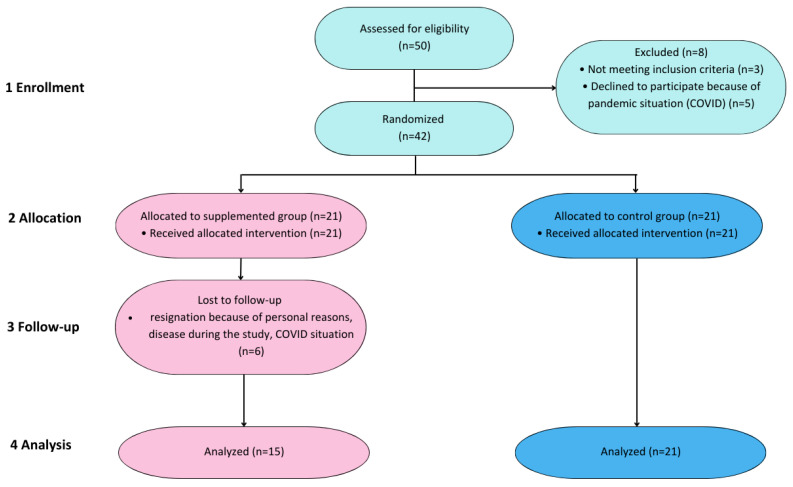
Flow diagram of the study.

The administration was planned for the autumn–winter season to prevent the confounding influence of sun exposure. The inclusion and exclusion criteria of PD patients and other relevant details were described by Bytowska et al. [14]. In total, 50 patients were recruited in the study. Three of them were excluded for not meeting the inclusion criteria and five were precluded by cause of resignation due to the COVID-19 pandemic. There were 42 patients who were randomized into VitD and PL groups. All patients (n = 21) from the PL group completed the intervention. Six patients from the VitD group resigned during the intervention due to the COVID-19 pandemic that progressed during the study, personal reasons, or illness during the study (Figure 1).

### 2.3. Intervention

The administration period was 12 weeks. The dosage of vitamin D_3_ was based on the patient’s BMI as follows: for normal weight (BMI below 25, 4000 International Units (IU)/day), for overweight (BMI 25–30, 5000 IU/day), and for obesity (BMI above 30, 6000 IU/day). Patients from the PL group received a matching placebo treatment.

### 2.4. Visit Programme and Material Collection

Patients were asked to meet with the investigators three times over the aforementioned administration period. The baseline visit (T0) included blood collection, signing of the consent forms, and the distribution of the administration bottles. Over the course of the second meeting, conducted six weeks after the start of the study, the administration bottles were refilled, and the dosage was corrected if necessary. The administration bottles were sent to patients unable to physically attend the second meeting. During the third meeting (T1), conducted 12 weeks after the start of the study, blood was drawn again. Telephone contact was maintained with patients between the meetings.

Blood was drawn into test tubes with a clot activator, then centrifuged at 2000× *g* for 10 min at 4 °C. The received serum samples were divided and stored at −80 °C until the analysis.

### 2.5. Measurement of Vitamin D and Kynurenine Pathway Metabolites

The vitamin D profile and kynurenine pathway were measured in serum by the mass spectrometry method. We estimated the concentration of 25(OH)D_3_, 24,25(OH)_2_D_3_, 3-HK, KYN, KYNA, XANA, and PA. The isotope dilution method by liquid chromatography combined with tandem mass spectrometry technique (LC-MS/MS) was applied. All samples were set up and analyzed with the Eksigent ExionLC analytical HPLC system with a CTC PAL autosampler (CTC Analytics AG, Zwingen, Switzerland) coupled with QTRAP^®^ 4500 MS/MS system (Sciex, Framingham, MA, USA).

### 2.6. Assessment of Inflammation Markers

The assessment of IL-6 was made by using an R and D Human IL-6 Quantikine HS ELISA Kit (HS600C, R&D Systems, Inc., Minneapolis, MN, USA) according to the manufacturer’s instructions. The measurement of TNF-α was made by using a Biorbyt Human TNF alpha ELISA Kit (orb50111, Biorbyt Ltd., Cambridge, United Kingdom) according to the manufacturer’s instructions. All of the samples were analyzed in a Thermo Scientific Varioskan LUX multimode microplate reader (Thermofisher Scientific, Vartaa, Finland).

### 2.7. Statistical Analysis

For the analysis, the statistics program Statistica 13 was used. Only complete data, from patients who finished all interventions (n = 36), were taken under consideration. The Shapiro–Wilk W-test was used to proofread data for normality. The descriptive statistics for both background information and examination of the trends in the analyzed parameters with mean values and 95% confidence intervals were used. The ANOVA test for repeated measures and the one-way ANOVA test were applied for the statistical analysis. Spearman rank correlation was calculated. The statistical significance was established at *p* < 0.05. To determine statistical significance, we used the post hoc Fisher’s Least Significant Difference test.

## 3. Results

### 3.1. Demographical Characteristics

Fifty patients were recruited in the study; thirty-six completed the intervention. All patients were Caucasian and fulfilled the inclusion criteria. No statistically relevant changes were detected among the groups. The mean age was 64 ± 9 years in the VitD group and 65 ± 6 years in the PL group. There were eight men (M) and seven women (W) in the VitD group and 17 M and four W in the PL group. The average body mass and height were correspondingly 78 ± 12 kg and 169 ± 11 cm in the VitD group and 80 ± 20 kg and 169 ± 15 cm in the PL group. The Hoen and Yahr (H and Y) score was 2.5 in both groups; the duration of the disease was between eight and thirteen years in the VitD and the PL group. DBS implantation in both groups was performed 3–5 years ago. The patients’ characteristics are shown in Table 1.

### 3.2. Kynurenine Pathway

**Figure 2:**
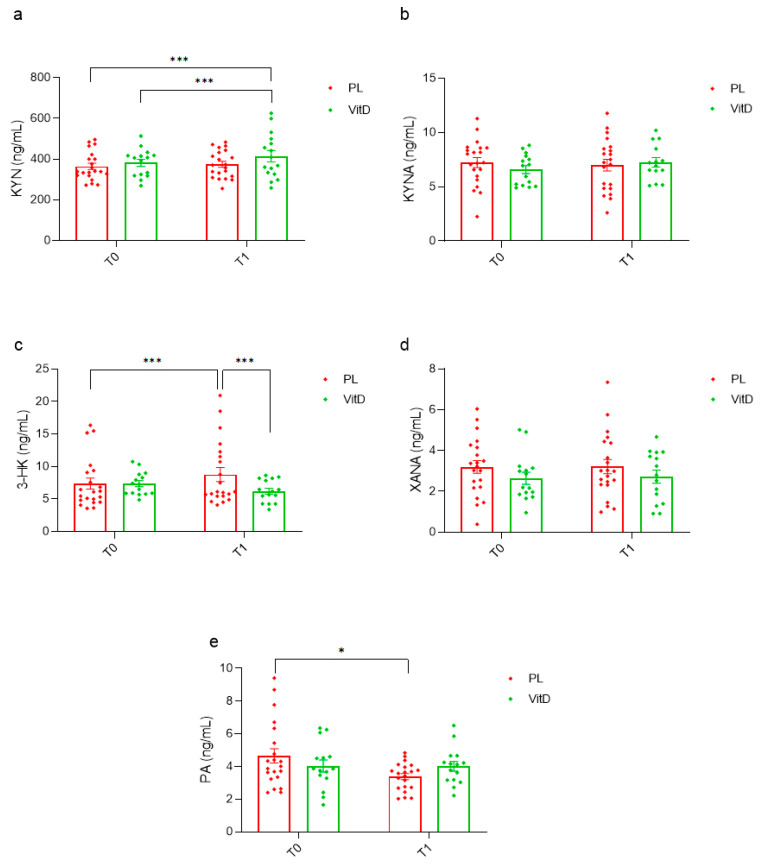
The impact of vitamin D_3_ administration on kynurenine pathway metabolites. (**a**) Changes in serum concentration of KYN. (**b**) Changes in serum concentration of KYNA. (**c**) Changes in serum concentration of 3-HK. (**d**) Changes in serum concentration of XANA. (**e**) Changes in serum concentration of PA. * *p* < 0.0006, *** *p* < 0.05, T0—before the research, T1—after 12 weeks of administration, VitD—vitamin D group, PL—placebo group, KYN—kynurenine, KYNA—kynurenic acid, 3-HK—3-hydroksykynurenine, XANA—xanthurenic acid, PA—picolinic acid, whiskers refer to standard error (SE).

The effect of vitamin D_3_ administration on the kynurenine pathway is presented below. KYN concentration increased significantly in the VitD group during the administration (382.14 ± 68.34 versus 422.17 ± 109.61 ng/mL, *p* < 0.05; Figure 2a). Additionally, a relevant difference between the PL group at T0 and the VitD group at T1 (364.54 ± 69.83 versus 422.17 ± 109.61 ng/mL, *p* < 0.05; Figure 2a) was noted. There were no significant alterations in KYNA concentrations observed in both groups. However, although an increasing trend in the VitD group was observed (6.60 ± 1.42 versus 7.29 ± 1.58 ng/mL, *p* = 0.067; Figure 2b), there was no notable difference in the PL group (7.23 ± 2.10 at T0, 7.00 ± 2.43 ng/mL at T1; Figure 2b). A statistically higher 3-HK concentration was found in the PL group 7.38 ± 3.92 at T0 versus 8.78 ± 4.93 ng/mL at T1 (*p* < 0.05; Figure 2c). Moreover, 3-HK differed significantly at T1 between groups: 6.23 ± 1.73 in the VitD group versus 8.78 ± 4.93 ng/mL the PL group (Figure 2c). There was no notable difference in XANA concentrations in the VitD group (2.64 ± 1.15 at T0, 2.72 ± 1.25 ng/mL at T1) and in the PL group (3.19 ± 1.43 at T0, 3.21 ± 1.60 ng/mL at T1) (Figure 2d). The PA concentration decreased significantly in the PL group (4.66 ± 2.03 compared to 3.38 ± 0.84 ng/mL, *p* < 0.0006; Figure 2e). In the VitD group, no differences were observed (4.03 ± 1.43 at T0, 4.01 ± 1.14 ng/mL at T1; Figure 2e).

**Figure 3:**
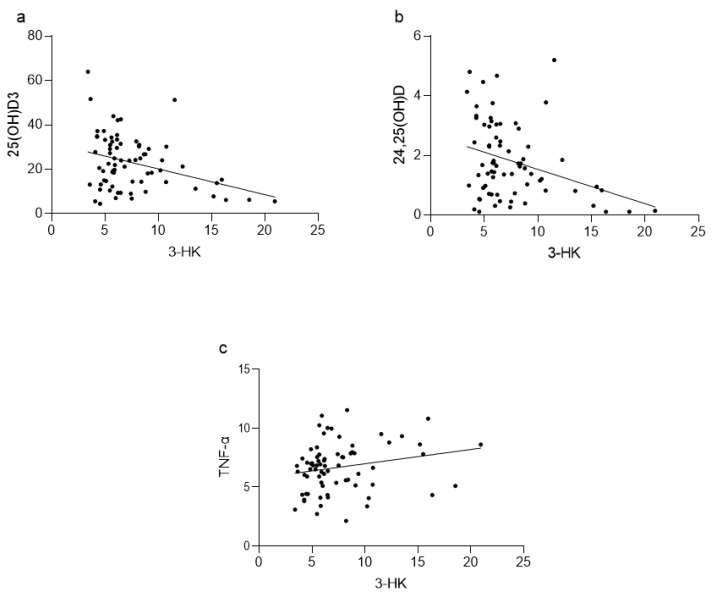
Correlations among (**a**) 25(OH)D_3_ and 3-HK; (**b**) 24,25(OH)_2_D_3_ and 3-HK, and (**c**) TNF-α and 3-HK. (**a**) Spearman r = −0.2421, *p* < 0.05, 95% confidence interval: −0.45 to −0.004; (**b**) Spearman r = −0.26, *p* < 0.05, 95% confidence interval: −0.47 to −0.03, (**c**) Spearman r = 0.2530, *p* < 0.05, 95% confidence interval: 0.01571 to 0.4633. KYNA—kynurenic acid, 3-HK—3-hydroksykynurenine, TNF-α—tumour necrosis factor-alpha.

A negative correlation between 25(OH)D_3_ and 3-HK (*p* < 0.05; Figure 3a), and between 24,25(OH)_2_D_3_ and 3-HK (*p* < 0.05; Figure 3b), was found. Furthermore, a positive correlation between TNF-α and 3-HK (*p* < 0.05; Figure 3c) was noticed.

**Figure 4:**
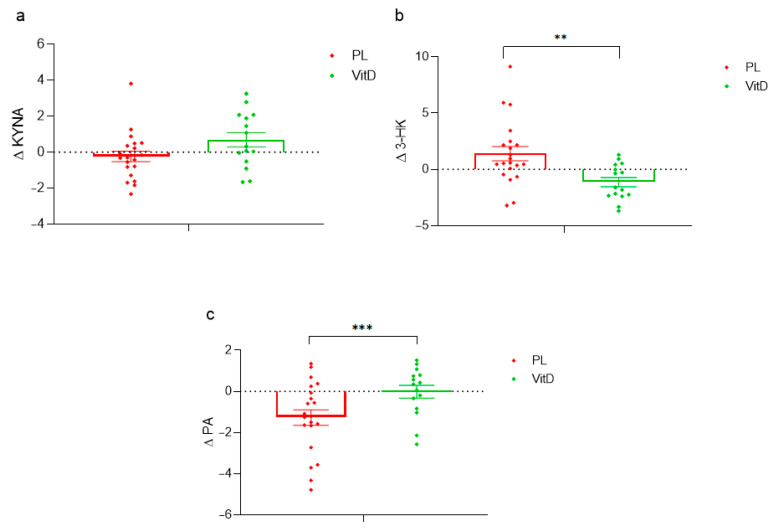
The effect of supplementation on Δ in KYNA, 3-HK, and PA between VitD and PL group. (**a**) Difference of Δ in KYNA. (**b**) Difference of Δ in 3-HK. (**c**) Difference of Δ in PA. ** *p* < 0.005, *** *p* < 0.05, Δ—delta, VitD—vitamin D group, PL—placebo group, KYNA—kynurenic acid, 3-HK—3-hydroksykynurenine, PA—picolinic acid, whiskers refer to standard error (SE).

Additionally, the differences in the delta of KYNA, 3-HK, and PA were examined between groups. In KYNA there was no significant difference, but a strong trend *p* = 0.062 (0.69 ± 1.54 in the VitD group compared to −0.23 ± 1.31 in the PL group; Figure 4a) was observed. The delta of neurotoxic 3-HK was relevantly downregulated in the VitD group as compared to the PL group (−1.14 ± 1.5, 1.40 ± 2.88, respectively; *p* < 0.005; Figure 4b). The delta of the neuroprotective metabolite of KP, PA, was statistically relevantly reduced in the PL group as compared to the VitD group (−1.27 ± 1.74, −0.01 ± 1.20, respectively; *p* < 0.05; Figure 4c) in PD patients with DBS.

### 3.3. Inflammation Status

**Figure 5:**
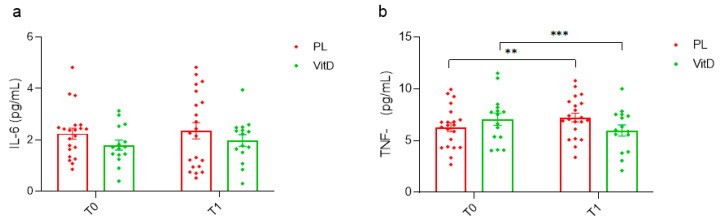
The impact of vitamin D_3_ administration on inflammation markers. (**a**) Changes in serum concentration of IL-6. (**b**) Changes in serum concentration of TNF-α. ** *p* < 0.005, *** *p* < 0.05, T0—before the research, T1—after 12 weeks of administration, VitD—vitamin D group, PL—placebo group, IL-6—interleukin 6, TNF-α—tumour necrosis factor-alpha, whiskers refer to standard error (SE).

The changes in inflammatory markers concentration after vitamin D_3_ supplementation are presented in Figure 5. No meaningful differences in IL-6 serum concentration (Figure 5a) were observed. In the VitD group the value was 1.80 ± 0.75 at T0 and 1.98 ± 0.87 pg/mL at T1, and in the PL group 2.24 ± 0.97 at T0 and 2.35 ± 1.45 pg/mL at T1. In TNF-α, a statistically marked reduction in the VitD group (7.07 ± 2.30 versus 5.98 ± 2.09 pg/mL, *p* < 0.05; Figure 5b) was found. Moreover, there was a statistically relevant increase in the PL group (6.30 ± 2.00 compared to 7.23 ± 1.94 pg/mL, *p* < 0.005; Figure 5b).

## 4. Discussion

Recently, we reported that a 12-week BMI-based vitamin D_3_ administration improved physical performance in patients with PD who underwent DBS. Moreover, the overall inflammatory state measured with high sensitivity C-reactive protein after the administration showed a decreasing trend [14]. In the current study, our goal was to expand our research and investigate whether BMI-based vitamin D_3_ supplementation in DBS PD patients can modify the KP and its impact on selected inflammatory markers. We found that after vitamin D_3_ administration based on the patient’s BMI, the serum concentration of TNF-α decreased in PD patients with DBS. Furthermore, we observed in KP that the neurotoxic substance 3-HK was enhanced in the PL group, the neuroprotective metabolite PA decreased in the PL group, and another neuroprotective metabolite KYNA tended to be higher after supplementation in patients with PD who underwent DBS. We also discovered that the delta of KYNA and PA were upregulated, and the delta of 3-HK was downregulated in the supplemented group. In PA and 3-HK the difference in delta between the groups was statistically significant, and in KYNA we observed a strong trend between groups. Furthermore, we not only noticed a negative correlation between 25(OH)D_3_, 24,25(OH)_2_D_3_, and 3-HK but also a positive correlation between TNF-α and 3-HK. Our results provide further evidence concerning a key link between the KP substances, inflammatory states, and metabolites of vitamin D in PD patients with DBS. Overall, the gathered data suggest that both vitamin D deficiency and dysregulation of KP metabolites are closely associated with PD development and progression. Our initial findings indicate that the vitamin D-kynurenine pathway may be associated with the pathogenesis of PD. Consequently, this approach can potentially offer a novel therapeutic strategy that does not solely target dopamine metabolism.

The activation of KP is primarily driven by the liver, and the majority of its metabolites originate from peripheral tissues. However, under specific physiological conditions such as aging or certain pathological conditions such as blood–brain barrier (BBB) damage, these metabolites have the potential to cross the BBB and affect the central nervous system. Moreover, it is also known that the KP is often disturbed in PD. Substances from KP such as 3-HK and XANA are known to be neurotoxic, whereas KYNA and PA are usually characterized as neuroprotective. In the literature, it was reported that during states of elevated inflammation, there is a diversion of tryptophan away from serotonin production toward the KP [16,23,24,25]. In the present study, we observed a relevant increase in KYN serum concentration in the VitD group after vitamin D_3_ administration. Our results are in line with a study conducted in 2017 [26], where the authors demonstrated higher KYN concentrations in patients with PD compared with healthy controls. However, other results are in contrast—lower KYN concentrations were observed in PD patients than in the control group [27]. KYNA concentrations are lower in PD patients than in healthy controls [27,28,29]. Nonetheless, some authors reported increased concentrations of KYNA in PD [26]. This indicates that the results are inconsistent among researchers of PD. However, it is difficult to consider whether our results are consistent with or contrary to those reported by others because our PD patients were treated with DBS. We are very far from any speculation, but stabilization of L-dopa dosage resulting from DBS treatment might potentially influence the concentration of KP metabolites. In our study, we did notice a trend toward increasing KYNA concentration in the VitD group (*p* = 0.067). 3-HK concentrations are often elevated in PD patients [24,30] suggesting that the KP in PD is more likely to play a neurotoxic rather than neuroprotective role during disease progression. Kynurenine-3-monooxidase which is responsible for converting KYN to 3-HK can be activated by inflammatory factors [16]. Our results showed a significant increase in the 3-HK concentration in the PL group whereas after administration in the VitD group, it remained at comparable levels. Moreover, after the administration, the 3-HK concentration was significantly lower in the VitD group than in the PL group. Furthermore, we found a negative correlation between 3-HK and 25(OH)D_3_ and 3-HK and 24,25(OH)_2_D_3_. Although the KYN concentration was relevantly lower in the PL group, the concentration of neurotoxic 3-HK was higher in this group. It might be an effect of the neuroprotective role of vitamin D_3_. Another metabolite that we measured was PA, which plays a neuroprotective role. The concentration of PA significantly decreased in the PL group.

Inflammation that is connected with age may stimulate the activation of KP. Moreover, the expression of IDO, which is the first enzyme of KP, can be influenced by inflammatory cytokines such as IL-6 and TNF-α [31,32]. This may reflect an increased risk of neurodegenerative disorders. In a review from 2016, researchers found that IL-6 and TNF-α concentrations were higher in PD patients than in healthy controls [20]. Xiromerisiou et al. showed that in the advanced stages of the disease (H and Y > 2) concentration of TNF-α is elevated [33]. Depression is a common non-motor symptom in PD. It was reported that TNF-α concentrations in the cerebrospinal fluid of depressive PD patients are higher than those in PD patients without depressive symptoms [34]. After the intervention, we found a marked reduction in TNF-α serum concentration in the VitD group and a relevant increase in the PL group. Furthermore, an increased production of the neurotoxic metabolite 3-HK may be triggered by inflammatory factors. The higher concentration of 3-HK was associated with higher TNF-α in the PL group. We found that these parameters are correlated. Our preliminary data indicate that vitamin D plays a neuroprotective role, particularly in reducing the concentration of 3-HK. This effect is notable when the concentrations of serum 25(OH)D_3_ reach optimal levels. In our previous study, we found that administration attenuated the general inflammation status, although the effect was not significant [14].

The study has some limitations. Firstly, the sample size is small because the recruitment of PD patients during the pandemic was impeded. Secondly, we did not measure the tryptophan concentrations but the kynurenine pathway is described as the main catabolism pathway (>95%) of tryptophan [16]. Thirdly, we did not have any food questionnaires, although patients claimed that they did not change their diet habits during the study.

## 5. Conclusions

In the current research, we observed a decrease in the concentration of the inflammatory marker TNF-α after vitamin D_3_ administration in PD patients with DBS. Furthermore, we found that the neurotoxic metabolite of KP, 3-HK, increased in the PL group, while the neuroprotective metabolite, PA, decreased in the same group. Interestingly, there was a tendency for the neuroprotective metabolite KA to be higher in the VitD group. Taken together, our findings may reflect the neuroprotective abilities of vitamin D_3_ in patients with PD who underwent DBS. The deficiency of vitamin D and disturbances in the kynurenine pathway are closely connected with the development of PD. Our preliminary data strongly support the notion that vitamin D, in conjunction with the kynurenine pathway, may be associated with the pathogenesis of PD.

## Figures and Tables

**Table 1:** Patients’ characteristics.

	VitD Group (n = 15)	PL Group (n = 21)
Age	64 ± 9 years	65 ± 6 years
Sex	8 M, 7 W	17 M, 4 W
Height	169 ± 11 cm	169 ± 15 cm
Body mass	78 ± 12 kg	80 ± 20 kg
H&y	2.5	2.5
Duration of the disease	8–13 years	8–13 years
Time from DBS implantation	3–5 years	3–5 years
25(OH)D_3_ T0 (ng/mL)	24.25 ± 9.23	18.99 ± 10.95
25(OH)D_3_ T1 (ng/mL)	34.12 ± 10.75	18.38 ± 11.98
24,25(OH)_2_D_3_ T0 (ng/mL)	2.05 ± 1.25	1.43 ± 1.11
24,25(OH)_2_D_3_ T1 (ng/mL)	2.85 ± 1.02	1.31 ± 1.21
BMI	≤25→4	≤25→8
	25–30→8	25–30→10
	≥30→3	≥30→3

## Data Availability

The data sets used and/or analyzed during the current study are available from the corresponding author upon reasonable request.
